# Serum CA19-9 response as a surrogate for clinical outcome in patients receiving fixed-dose rate gemcitabine for advanced pancreatic cancer

**DOI:** 10.1038/sj.bjc.6602687

**Published:** 2005-07-05

**Authors:** A H Ko, J Hwang, A P Venook, J L Abbruzzese, E K Bergsland, M A Tempero

**Affiliations:** 1 University of California at San Francisco Comprehensive Cancer Center, 1600 Divisadero Street, 4th Floor, Box 1705, San Francisco, CA 94115, USA; 2 MD Anderson Cancer Center, 1515 Holcombe Blvd., Houston, TX 77030-4009, USA

**Keywords:** pancreatic cancer, CA19-9, gemcitabine, tumour marker, surrogate end point

## Abstract

The use of serial serum measurements of the carbohydrate antigen 19-9 (CA19-9) to guide treatment decisions and serve as a surrogate end point in clinical trial design requires further validation. We investigated whether CA19-9 decline represents an accurate surrogate for survival and time to treatment failure (TTF) in a cohort of 76 patients with advanced pancreatic cancer receiving fixed-dose rate gemcitabine in three separate studies. Statistically significant correlations between percentage CA19-9 decline and both overall survival and TTF were found, with median survival ranging from 12.0 months for patients with the greatest degree of biomarker decline (>75%) compared with 4.3 months in those whose CA19-9 did not decline during therapy (*P*<0.001). Using specific thresholds, patients with ⩾25% decline in CA19-9 during treatment had significantly better outcomes than those who did not (median survival and TTF of 9.6 and 4.6 months *vs* 4.4 and 1.5 months; *P*<0.001). Similar results were seen using both 50 and 75% as cutoff points. We conclude that serial CA19-9 measurements correlate well with clinical outcomes in this patient population, and that decline in this biomarker should be entertained for possible use as a surrogate end point in clinical trials for the selection of new treatments in this disease.

Pancreatic cancer represents the fourth leading cause of cancer-associated mortality in the United States, with the annual mortality rate approximating the incidence rate (Jemal *et al*, 2005). Clinical trials of novel therapeutic agents in this disease often report response rate as a study end point, but the usefulness and accuracy of this outcome variable are debatable. First of all, the ability to monitor objective responses to systemic therapy in patients with advanced pancreatic cancer, particularly at the primary pancreatic site, can be difficult using conventional methods such as computerised tomography. Measurement of objective response by formal RECIST criteria does not allow one to gauge accurately the true burden of disease due to the extensive desmoplasia and surrounding inflammation associated with pancreatic tumours and the inability to trace clearly defined tumour margins of the primary pancreatic lesion (Rothenberg *et al*, 1996). Furthermore, promising response rates in early trials do not uniformly translate into significant improvement in patient outcomes when investigated in phase III studies. Hence, alternative methods for monitoring patients on therapy are critical for guiding early treatment decisions, offering prognostic information, and implementing into clinical trial design as new surrogate end points for the selection of therapeutic agents.

Carbohydrate antigen 19-9 (CA19-9) is the sialylated Lewis blood group antigen originally defined by the monoclonal antibody 1116 NS 19-9 (Koprowski *et al*, 1979, 1981). A radioimmunoassay was developed for this marker in 1983 (Del Villano *et al*, 1983), and since that time, measurement of CA19-9 levels in serum has been commonly used as an adjunct in the diagnosis of pancreatic cancer. Approximately three-quarters of all patients with pancreatic cancer have an elevated serum CA19-9 level at baseline (Yeo *et al*, 2002). Patients who are genotypically negative for the Lewis blood group antigen, however, will not express CA19-9 even in the presence of active pancreatic cancer. Using as a cutoff point of 37 U ml^−1^ as the upper limit of normal, overall sensitivity of the assay is approximately 80% and its specificity is 90% for detecting pancreatic cancer (Steinberg, 1990). Additionally, serial measurements of CA19-9 are frequently performed for prognostic purposes, for gauging disease relapse and activity, and for monitoring patients undergoing therapy as an approximate surrogate for response.

Despite the frequency of its use in clinical practice, application of the CA19-9 biomarker as a strategy to guide treatment decisions and to serve as a primary end point in clinical trial design has not been well established. In particular, correlation between marker decline during chemotherapy and patient outcomes requires further validation. This analysis investigated whether, and to what degree, CA19-9 decline represented an accurate prognostic test in a group of study patients with advanced pancreatic cancer undergoing treatment with gemcitabine administered at fixed-dose rate (FDR) infusion.

## MATERIALS AND METHODS

This retrospective analysis pooled data from three separate studies examining the role of gemcitabine administered at a FDR infusion in the treatment of advanced pancreatic cancer. These three studies included: (1) a randomised phase II study of gemcitabine given either by FDR infusion (10 mg^−1^m^−2^ min^−1^) at 1500 mg m^−2^ or by standard 30-min infusion at 2200 mg m^−2^, given on days 1, 8, and 15 of a 28-day cycle; (2) a phase I study of FDR gemcitabine at doses ranging from 1000 to 1200 mg m^−2^ plus cisplatin 20 mg m^−2^, both administered on days 1 and 8 of a 21-day cycle; and (3) a phase II study of FDR gemcitabine at 1000 mg m^−2^ plus cisplatin at 20 mg m^−2^, both administered on days 1 and 8 of a 21-day cycle (Table 1). Research support for these clinical trials was provided by Eli Lilly. For study #1, only patients on the FDR gemcitabine arm were eligible for inclusion in the current analysis, to maintain as much uniformity as possible in patient and treatment characteristics. A total of five different institutions enrolled patients in one or more of these three studies.

To be eligible for inclusion in this analysis, subjects were required to have a confirmed diagnosis of pancreatic adenocarcinoma (either locally advanced or metastastic), a baseline serum CA19-9 level greater than 75 U cm^−3^ (two-fold the upper limit of normal), no prior systemic therapy, and intact renal, hepatic, and haematologic function. The absence of clinical or laboratory evidence of biliary obstruction at baseline minimised the possibility that CA19-9 levels would be attributable to this cause rather than a true reflection of disease activity.

In each of these three clinical trials, the serum level of CA19-9 was measured at the start of each treatment cycle as part of the ongoing clinical evaluation, approximately once every 3–4 weeks. Given the retrospective nature of this study, with analysis of data from three separate trials incorporating several institutions, it was not possible to ascertain the uniformity of lab methodology between different patients, as a single central laboratory was not used. However, all serum CA19-9 measurements for any given patient were routinely performed at the same laboratory, ensuring some degree of intrapatient consistency. Patients with only one CA19-9 measurement were categorised as nondecliners; the most common reason for the lack of follow-up CA19-9 measurements in these patients was death and/or early disease progression requiring discontinuation of study treatment.

CA19-9 measurements over the entire course of treatment were recorded. Descriptive statistics were used to record baseline CA19-9 levels and the per cent change of this biomarker relative to baseline. Subjects were placed in different categories based on their level of biomarker decline achieved at any point throughout the course of treatment (0–25% decline, 26–50% decline, 51–75% decline, and 76–100% decline, as well as no decline) and by absolute threshold (greater than or less than 25% decline, 50% decline, and 75% decline). We did not require a sustained biomarker decline (i.e. confirmation by two measurements spaced at least 28 days apart) for this analysis.

*χ*^2^ tests were used to evaluate the statistical association between baseline CA19-9 categorised into subgroups and subsequent maximum per cent decline in biomarker decline. The relationships between biomarker responses with patient outcomes, including overall survival and time to treatment failure (TTF), and (where available) objective radiographic response were then analysed. The Kaplan–Meier product limit method was used to estimate the probability of survival and of remaining free of treatment failure accounting for censored observations with the median value used to summarise the results. For comparisons of median survival and TTF between defined subcategories, the Wilcoxon–Mann–Whitney rank-sum log-rank test was performed. The Kaplan–Meier method with log-rank test was used to evaluate significant differences in survival and TTF accounting for censored data. Cox's proportional-hazard model was used to determine whether CA19-9 was a significant predictor of both survival and TTF. Statistical correlation between changes in CA19-9 and clinical outcomes was confirmed using Spearman's rank correlation.

## RESULTS

### Patient characteristics

Clinical and CA19-9 data were examined from a total of 103 patients from the three clinical studies. Of these, 76 patients (73.8%) were eligible for inclusion in this study based on a pretreatment serum CA19-9 measurement greater than two-fold the upper limit of normal. Of the 76 eligible patients, 73 (96%) had suspected or documented extrapancreatic metastases. The median overall survival in this group of patients was 7.0 months (range, 0.2–35 months), and median TTF was 3.6 months (range, 0.2–29 months).

### CA19-9 measurements

The median baseline CA19-9 level was 3052 U cm^−3^ (range, 98–832 050 U cm^−3^) (Table 1). Patients had serum CA19-9 measurements approximately once per month, at the start of each treatment cycle. The number of serial measurements ranged from 1 to 17 (median number of measurements, 4.5). There was a fairly even distribution of degree of CA19-9 decline achieved at any point during therapy compared with baseline when broken down by degree of decline (0–25, 26–50, 51–75, and 76–100% decline) (Table 2). The absolute numbers in each group, however, were too small to determine any statistically significant difference in distribution between subsets. In all, 25% of patients exhibited no decline in their CA19-9 level at any point during therapy (Table 2). Seven patients had only a solitary CA19-9 measurement at baseline (and, per study definition, were classified as nondecliners). In terms of absolute thresholds of CA19-9 decline, 68.4% of patients achieved at least a 25% decline in CA19-9; 52.6% of patients achieved at least a 50% marker decline; and 31.6% of patients achieved at least a 75% marker decline (Table 3).

We also evaluated whether the baseline CA19-9 level had any effect on the likelihood or degree of subsequent biomarker decline by dividing subjects into quartiles according to their baseline values (Table 4). *χ*^2^ tests showed no significant association between baseline CA19-9 level and subsequent decline.

### Relationship between CA19-9 and patient outcomes

Strong, statistically significant differences in median survival and TTF were observed when patients were grouped by quartile according to the percentage of CA19-9 decline (*P*<0.001 for both survival and TTF) (Table 2). For example, patients with the greatest degree of decline (>75%) had a median survival and TTF of 12.00 and 6.00 months, respectively, compared to 6.02 and 3.36 months in patients with only a 0–25% decline. Patients who did not achieve any decline in CA19-9 at any point during the course of treatment fared the most poorly, with a median survival of 4.31 months and a median TTF of 1.35 months. This trend toward increasing survival held for each successive subset of biomarker decline. The same trend of greater percentage biomarker decline corresponding with improved clinical outcomes held true for median times to treatment failure, with the exception of the (0–25%) and (26–50%) subsets.

Additionally, we carried out sequential analyses examining correlation between CA19-9 decline and patient outcomes, this time using various thresholds of percentage decline as cutoff points. These analyses demonstrated statistically significant differences in both median survival and median TTF when comparing patients who fell above *vs* below thresholds of 25, 50, and 75% biomarker decline (Table 3). For example, using as a benchmark a 50% decline in CA19-9 (as is frequently reported in clinical trials), we found a fairly even distribution between those who achieved at least a 50% decline *vs* those who did not (52.6 *vs* 47.4%). Those with >50% decline had a median survival of 10.80 months and a median TTF of 4.93 months compared to 5.82 and 2.07 months in patients who did not have at least a 50% decline. Analysis of these data using Spearman's rank correlation confirmed the significant association between percentage CA19-9 change from baseline and both survival and the TTF (*r*=−0.345 (*P*=0.004) and *r*=−0.322 (*P*=0.007)).

Of note, 37 of the 76 patients (48.7%) demonstrated an early biomarker response, defined as a decline in CA19-9 levels by a minimum of 25% within the first two measurements after baseline (data not shown). Additionally, 23 patients (30.3%) had an early biomarker decline of at least 50%, and 11 (14.5%) showed at least a 75% early decline. Statistically significant differences in median survival when comparing patients achieving early biomarker response *vs* those who did not was found only when using the 25% threshold, although this is likely attributable to the small numbers of patients achieving early biomarker response using the 50 and 75% thresholds.

Objective response by RECIST criteria was not a defined end point of any of the trials from which these data were collected; however, unconfirmed responses were recorded as part of the record-keeping for trials 1 and 3. In total, 11 unconfirmed responses were tallied. Nine of these patients experienced a CA19-9 decline of 50–74%, and two patients experienced a CA19-9 decline of greater than 75%. Conversely, no patient with less than a 50% biomarker decline showed evidence of objective radiographic response.

We used Cox's multivariate proportional-hazard regression model to evaluate the impact of three separate factors on clinical outcomes: baseline CA19-9 concentration, the study the patient was treated on, and percentage CA19-9 decline. Of these, only percentage CA19-9 decline was strongly related to both survival and TTF (data not shown).

## DISCUSSION

A number of previous studies have provided justification for measuring CA19-9 levels in patients with advanced pancreatic cancer receiving either chemotherapy or radiation, both for prognostic and for monitoring purposes. Patients with higher CA19-9 levels prior to initiation of chemoradiation for locally advanced disease have poorer outcomes in terms of both response rates and overall survival (Ikeda *et al*, 2001; Micke *et al*, 2003b). A lower CA19-9 level following completion of chemoradiation also appears to correlate with survival (Micke *et al*, 2003a). Furthermore, the degree of change in CA19-9 levels during radiotherapy may indicate how well a patient will fare, with two separate studies demonstrating that either a 50 or a 75% biomarker decline correlates with improved median survival time (Katz *et al*, 1998; Okusaka *et al*, 1998).

With the widespread use of gemcitabine as the mainstay of treatment in patients with advanced pancreatic cancer, recent reports have begun to examine CA19-9 response to gemcitabine-based treatments and whether kinetics of CA19-9 can serve as a predictor of response to such treatments. A small retrospective study by Saad *et al* (2002) of 28 patients with advanced pancreatic cancer treated with gemcitabine found that lower pretreatment levels of CA19-9, as well as a ⩾50% decline in CA19-9 anytime during treatment, correlated with better survival rates. Halm *et al* (2000) examined 43 unresectable patients receiving gemcitabine treatment and found that those with a >20% decrease of their baseline CA19-9 level after 8 weeks of treatment had a longer median survival than those with a rise or a decrease <20% (268 *vs* 110 days), a finding confirmed by Ziske *et al* (2003). This biomarker response was in fact the only independent predictor of survival in a multivariate analysis, showing a greater level of significance than either objective tumour response or clinical benefit response (Ziske *et al*, 2003). Heinemann and colleagues, meanwhile, collected CA19-9 data from patients with advanced pancreatic cancer receiving treatment on a study protocol using a combination of cisplatin and gemcitabine (Stemmler *et al*, 2003). CA19-9 responders, defined as those with a ⩾50% decrease in CA19-9 levels within 2 months after the start of treatment, survived significantly longer than CA19-9 nonresponders (295 *vs* 174 days, *P*=0.022).

Our study represents the first to examine whether changes in CA19-9 correlate with clinical outcomes in patients with advanced pancreatic cancer treated with FDR gemcitabine. Infusion of gemcitabine at an FDR is a strategy intended to optimise pharmacokinetics of the drug (Grunewald *et al*, 1990). In a recently published randomised phase II study, Tempero *et al* (2003) reported that administration of gemcitabine by FDR infusion resulted in superior outcomes in patients with advanced pancreatic cancer compared to administration of this drug by standard 30-min infusion.

We were specifically interested in examining how changes in CA19-9 during treatment with FDR gemcitabine correlate with clinical outcomes. Our response criteria were purposely broad in that a decline from baseline in CA19-9 at any point in time after initiation of treatment, whether early or late, was counted as a biomarker response, although a significant proportion of patients who responded did so within the first 2 months of therapy. While we specifically did not require a sustained biomarker response for the purposes of this study, all but seven patients did have a sustained decline in CA19-9 confirmed over two consecutive measurements. Analysis of our data even recategorising those seven patients as nonresponders did not affect the strong correlations found in this study.

Additionally, seven patients (9.2%) had only a solitary CA19-9 measurement at baseline. While we recognise the inherent bias in including these patients in our analysis (follow-up measurements were generally not obtained on these patients because of rapid disease progression and/or clinical deterioration), we ultimately decided to count them as biomarker nondecliners. Again, reanalysis of our data if we excluded these patients did not affect our study results in any way. Based on the results of our analysis, a rising/nondeclining CA19-9 appears to be a clear indicator of early progressive disease and to correlate with very poor clinical outcomes. This finding may potentially be used in clinical practice as justification for discontinuing systemic therapy early on.

One limitation of our analysis was the lack of uniformity in treatment and in methodology for assaying CA19-9 levels between different patients, given the retrospective and multi-institutional nature of this analysis. However, we did intentionally select three trials in which gemcitabine was consistently administered by FDR infusion, and we believe that the addition of low-dose cisplatin in two of the three trials should not have any substantial impact on our findings. Additionally, each clinical trial did attempt to maintain intrasubject consistency in terms of the laboratory where each subject's serial CA19-9 levels were measured. In the future, clinical studies incorporating CA19-9 measurements should use a standardised laboratory assay to ensure reliable results. Ideally, approval of this test by the Food and Drug Administration for monitoring patients on systemic therapy would further enhance these efforts.

As all the clinical trials from which data were extracted did not include objective response as an end point, we were only able to use unconfirmed response data recorded by study investigators in two of the three trials to correlate CA19-9 responses to radiographic responses. Some might argue that objective responses are the truest indicator of therapeutic activity. Nonetheless, the outcome variables we chose for this study, particularly overall survival, are the most relevant and clinically meaningful in terms of deciding whether a particular agent is worthy of further study or approval. We chose TTF rather than time to time to tumour progression as the other major outcome variable to examine because a number of patients on these trials discontinued study treatment for reasons other than disease progression; thus, censoring these data at those time points would have diluted our numbers substantially.

This analysis demonstrates that declines in serum CA19-9 levels of at least 25, 50, and 75% during treatment with a FDR gemcitabine-containing regimen all correspond with improved patient outcomes. Despite the relatively small numbers in this analysis, the highly statistically significant findings indicate a strong correlation between CA19-9 response and both survival and TTF. When clinical trials report biomarker response data, they generally use 50% as the threshold as an indicator of success. Our analysis suggests that a 25% decline in CA19-9 may provide adequate evidence for the clinical efficacy of a new therapeutic agent or combination treatment strategy. Furthermore, grouping the biomarker response by quartile demonstrates that greater declines in CA19-9 are associated with improved clinical outcomes. While this may be an intuitively obvious concept, ours is the first study to provide conclusive evidence to support a clear and direct correspondence between the degree of biomarker decline and how well patients fare with their disease.

In conclusion, serial CA19-9 measurement represents a useful prognostic tool in patients receiving chemotherapy for advanced pancreatic cancer, with at least a 25% threshold in decline from baseline correlating well with improved patient outcomes. While our analysis was limited to patients receiving an FDR gemcitabine-based regimen, there is little reason to suspect that our findings would not be broadly applicable to other systemic therapies. Thus, monthly CA19-9 measurements appear to be justified for predicting outcomes to therapy and tailoring treatment decisions, and should be considered as a surrogate end point in clinical trials for the selection of new treatments.

## Figures and Tables

**Table 1:** Baseline serum CA19-9 values of subjects in each study

**Study description**	**Dates of enrollment**	**No. of subjects eligible for this analysis**	**Median baseline CA19-9 (U cm^−3^)**	**Baseline CA19-9, range (U cm^−3^)**
Randomised phase II study of FDR gemcitabine *vs* standard infusion gemcitabine	1996–1999	25^a^	3400	98–263 000
				
Phase I study of FDR gemcitabine+cisplatin	1999–2000	10	3050	136–60 000
				
Phase II study of FDR gemcitabine+cisplatin	2001–2004	41	2729	129–832 050
				
Total		76	3052	98–832 050

CA19-9=carbohydrate antigen 19-9; FDR=fixed-dose rate infusion of 10 mg m^−2^ min^−1^.

a To maintain as uniform standards of treatment as possible across the three studies, only patients on the FDR arm were included for this analysis.

**Table 2:** Analysis by CA19-9 subgroup

**Group**	**Number of patients**	**Median CA19-9 decline, absolute value (U cc^−3^) (95% CI)**	**No. of pts. censored for survival**	**Median survival (mos) (95% CI)**	**No. of pts. censored for TTF**	**Median time to treatment failure (mos) (95% CI)**
No decline in CA19-9	19 (25.0%)	N/A	1 (5.3%)	4.31 (3.00–5.20)	1 (5.3%)	1.35 (1.00–2.76)
0–25% decline	5 (6.6%)	114.0 (83–5500)	0	6.02 (3.80–9.67)	0	3.36 (1.38–5.72)
26–50% decline	12 (15.8%)	1424.0 (26–55 000)	0	7.04 (5.16–10.00)	3 (25.0%)	3.60 (2.07–4.14)
51–75% decline	16 (21.1%)	2751.2 (160–279 074)	2 (12.5%)	7.55 (5.16–10.80)	2 (12.5%)	3.80 (2.24–4.44)
>75% decline	24 (31.6%)	3251.5 (236–263 490)	2 (8.3%)	12.00 (9.21–13.59)	5 (20.8%)	6.00 (4.87–14.50)
						
Log-rank *χ*^2^				31.20 (df=4, *P*<0.001)		50.34 (df=4, *P*<0.001)

CA19-9=carbohydrate antigen 19-9; CI=confidence interval; mos=months; TTF=time to treatment failure; df=degree of freedom.

**Table 3:** Analysis using specific thresholds for CA19-9 decline (reported results account for censored patients)

	**Yes**	**No**	**Log-rank** ***χ*2**
*>25% decline in CA19-9?*			
Number of patients (%)	52 (68.4%)	24 (31.6 %)	
Median survival (mos) (95% CI)	9.61 (8.00–11.50)	4.64 (3.45–5.82)	24.07 (df=1, *P*<0.001)
Median TTF (mos) (95% CI)	4.38 (3.88–6.00)	1.51 (1.00–2.76)	33.78 (df=1, *P*<0.001)
			
*>50% decline in CA19-9?*			
Number of patients (%)	40 (52.6%)	36 (47.4%)	
Median survival (mos) (95% CI)	10.80 (9.00–12.00)	5.82 (3.31–6.64)	14.58 (df=1, *P*<0.001)
Median TTF (mos) (95% CI)	4.93 (4.00–6.15)	2.07 (1.41–3.36)	26.20 (df=1, *P*<0.001)
			
*>75% decline in CA19-9?*			
Number of patients (%)	24 (31.6%)	52 (68.4%)	
Median survival (mos) (95% CI)	12.00 (9.21–13.59)	6.00 (5.00–6.81)	16.20 (df=1, *P*<0.001)
Median TTF (mos) (95% CI)	6.00 (4.87–14.50)	2.76 (1.80–3.60)	21.13 (df=1, *P*<0.001)

CA19-9=carbohydrate antigen 19-9; CI=confidence interval; mos=months; TTF=time to treatment failure; df=degree of freedom.

**Table 4:** Likelihood of CA19-9 decline as a function of baseline CA19-9 value

		**Number of subjects with change in CA 19-9(%)**					
**Baseline CA19-9 level**	**Range (U cc^−3^)**	**No decline**	**0–24% decline**	**25–49% decline**	**50–74% decline**	**75–100% decline**	**Total**
Lowest quartile	75–972	5 (26.3%)	3 (15.8%)	4 (21.1%)	3 (15.8%)	4 (21.1%)	19
2nd quartile	973–3052	6 (31.6%)	1 (5.3%)	2 (10.5%)	4 (21.1%)	6 (31.6%)	19
3rd quartile	3053–12 815	4 (21.1%)	0 (0.0%)	2 (10.5%)	5 (26.3%)	8 (42.1%)	19
Highest quartile	>12 815	4 (21.1%)	1 (5.3%)	4 (21.1%)	4 (21.1%)	6 (31.6%)	19
							
Total		19	5	12	16	24	76

CA19-9=carbohydrate antigen 19-9.

The *χ*^2^ test shows that there is no significant association between baseline CA19-9 and likelihood or degree of marker decline (*χ*^2^=7.556, *P*=0.82).
